# Contextual factors in shared decision making: a randomised controlled trial in women with a strong suspicion of breast cancer

**DOI:** 10.1038/sj.bjc.6604916

**Published:** 2009-02-10

**Authors:** A Vodermaier, C Caspari, J Koehm, S Kahlert, N Ditsch, M Untch

**Affiliations:** 1 Department of Obstetrics and Gynaecology – Grosshadern, University of Munich, Marchioninistr. 15, Munich 81377, Germany; 2 Department of Psychology, University of British Columbia, West Mall, Vancouver, Canada BC V6T 1Z4; 3 Helios Klinikum Berlin Buch, Gynecological Clinic, Breast Cancer Center, Schwanebecker Chaussee 50, Berlin 13125, Germany

**Keywords:** decision aids, intervention, breast cancer inpatients, decisional conflict, shared decision making, information

## Abstract

Decision aids in North American breast cancer outpatients have been shown to assist with treatment decision making and reduce decisional conflict. To date, appropriate delivery formats to effectively increase patient participation in newly diagnosed breast cancer inpatients have not been investigated in the context of German health care provision. The impact of a decision aid intervention was studied in patients (*n*=111) with a strong suspicion of breast cancer in a randomised controlled trial. The primary outcome variable was decisional conflict. Participants were followed up 1 week post-intervention with a retention rate of 92%. Analyses revealed that the intervention group felt better informed (*η*_p_^2^=0.06) but did not experience an overall reduction in decisional conflict as compared with the control group. The intervention had no effect on uptake rates of treatment options, length of consultation with the surgeon, time point of treatment decision making, perceived involvement in decision making, neither decision related nor general patient satisfaction. Patients who received the decision aid intervention experienced a small benefit with regards to how informed they felt about advantages and disadvantages of relevant treatment options. Results are discussed in terms of contextual factors and individual differences as moderators of treatment decision aid effectiveness.

Patient involvement in treatment decision making has been increasingly advocated in oncology. Many patients with breast cancer, however, do not feel adequately involved in treatment decision making (Bruera *et al*, 2002; Keating *et al*, 2002; Janz *et al*, 2004) and experience communication barriers with physicians (Sepucha *et al*, 2002).

Although most intervention studies on shared decision making have been completed in Canada and the United States, the impact of this approach in European health care systems remains unclear. Existing patriarchal physician attitudes and a more hierarchical health care system in Germany may make it more difficult to implement shared decision making into oncology health care provision. These specifics within the health care system may have created a culture of doctor–patient interaction that can complicate the implementation of shared decision making. In addition, the treatment of cancer patients is routinely offered in an inpatient setting in specialised centres or programs which may influence the efficacy of interventions, most likely in an impeding way, as inpatient care usually results in more passive patient behaviour, which in turn is less likely to be compatible with a shared decision making approach.

Despite these hypothesised hindrances and with the aim to develop new models of care, the German Ministry of Health announced a focus programme in 2001 to start research in the area of shared medical decision making. The current study reports on a model project of breast cancer treatment within this framework.

Given the importance of surgical and chemotherapy treatment for patients' quality of life (Moyer, 1997; Mols *et al*, 2005; Nelson *et al*, 2007) and that some treatment alternatives exist, sensitive decision making accounting for the patients' preferences should be the ultimate goal for routine care. To achieve this aim, decision aids have been developed as a tool to facilitate patient involvement in treatment decision making. Decision aids provide patients with disease-specific knowledge and assist in the recognition and the appreciation of their personal values to achieve informed decisions. Across diseases, decision aid interventions have demonstrated effects on patient knowledge, decisional conflict, concordance rates between values and the option chosen, and on patients' active role in decisions (O'Connor *et al*, 2003).

Although not all studies showed benefits (Goel *et al*, 2001), intervention studies in patients with newly diagnosed breast cancer found that decision aids increase patient knowledge (Sawka *et al*, 1998; Whelan *et al*, 2003, 2004) and affect surgical (Street *et al*, 1995; Molenaar *et al*, 2001; Katz *et al*, 2005; Lantz *et al*, 2005) as well as adjuvant treatment decision making (Peele *et al*, 2005; Siminoff *et al*, 2006).

Although several studies explored the effect of decision aid interventions on treatment decision making as a proxy for the effectiveness of the intervention, we believe that patient attitudes toward the decision making process may be a more important indicator of shared decision making effectiveness as the ultimate treatment choice may depend on several individual factors. Of these, decisional conflict has been demonstrated to be a sensitive measure of decision aid effectiveness (O'Connor *et al*, 2003).

To date, all randomised controlled trials in women with breast cancer have been conducted in North America. Therefore, the aim of this study was to examine the impact of a decision aid intervention in breast cancer inpatients in Germany. Because of the predominance of the inpatient treatment setting, we posited that decision aid interventions may have different, and presumably fewer effects due to the shorter time frame for decision making and the loss of a certain degree of patient autonomy from hospital admission. Therefore, we wanted to test whether or not effects on decisional conflict and higher patient information status could be replicated in this context. Consequently, experienced decisional conflict was considered the primary outcome variable.

We were also interested in whether or not the intervention may have changed the doctor—patient encounter with greater patient activation by the physician, more information seeking by the patient, or whether or not the intervention lead to more time needed for the consultation. It was expected that the intervention would encourage a more active patient role including more questioning behaviour or alternatively, the intervention may have clarified questions that the senior physician may have otherwise been asked, therefore, reducing patient participation in the consultation.

We further expected that the decision aid intervention might delay treatment decision making, with more patient involvement resulting in longer periods of time to consider potential treatment options.

Although essentially null effects were reported in a meta-analysis of intervention effects on satisfaction measures (O'Connor *et al*, 2003), some studies of breast cancer patients found positive effects of decision aid interventions (Sawka *et al*, 1998; Whelan *et al*, 2004). Consequently, we assessed whether the intervention influenced either decision specific or overall patient satisfaction with treatment.

Finally, we assessed the type of treatment decision making. We did not expect systematic differences in decision making by the treatment arm. At each of the stages examined, patients could choose from two treatment options, each possessing specific advantages as well as unique problems as patients weigh benefits and side effects of treatment choices differently. For example women undergoing breast-conserving therapy have generally better body image outcomes but need radiation therapy in addition to surgery with its long-term side effects on well-being. These women also have a higher risk of recurrence, whereas patients who opt for mastectomy may have ‘peace of mind’ but are more likely to experience body image disruptions and sexual problems. Although women with early-stage breast cancer who undergo adjuvant chemotherapy in addition to hormonal therapy may feel more secure with regards to tumour recurrence, they are at risk of long-term decrements in quality of life including cognitive impairment whereas patients who decide against chemotherapy may feel anxious not having tapped into the full potential of oncology treatment. Women who decide on preoperative chemotherapy choose a treatment regimen for which no long-term results are available at the time of trial conduction but value breast-conserving therapy as very important whereas those who decide on standard treatment receive a treatment that has been better studied.

## Patients and methods

### Sample

We recruited patients with a strong suspicion of having breast cancer from the gynaecological department of the University of Munich-Grosshadern. A potential breast cancer diagnosis was derived from extensive diagnostic procedures (mammogram, ultrasound, MRI). In addition, routine breast biopsy was conducted for patients with T2 or T3 tumour size as measured by ultrasound. Patients were identified in an inpatient setting and asked if they would be willing to participate in the study by two research psychologists. Eligible patients (a) had histologically proven breast cancer or strong suspicion through diagnostic procedures, (b) had an operable finding (tumour sizes T1, T2, or T3 as measured by ultrasound), (c) were between ages 18 and 75 years, (d) had no major psychiatric disease, dementia or mental retardation, (e) had good knowledge of the German language and (f) provided informed consent to the study. Patients were excluded if they had (a) a previous history of breast cancer (and *in situ* breast cancer) or other malignant tumours (except cured carcinoma *in situ*), (b) Stage IV breast cancer, (c) a medical contraindication for breast-conserving therapy or radiation in patients with tumour size T1, (d) co-morbidities that resulted in a distinct treatment option, leaving no range for choice, such as pregnancy and lactation.

### Study design

A two-arm, randomised controlled trial with decision aids *vs* standard treatment was conducted. Randomisation was conducted after the patient gave written informed consent to participate in the trial. Blinding was not possible within the hospital procedures. Nonetheless, in most cases physicians did not know in which arm patients had been randomised. Patients were assessed pre-randomisation (baseline) and at 1 week follow-up. The baseline questionnaire was filled out shortly after hospital admission and prior to randomisation. A few hours later, the intervention group received a 20-min decision aid intervention and additional written information, whereas the control group received standard care. For completion of the follow-up, patients received a pre-paid envelope to send back the questionnaire. The study was approved by the ethics committee of the University of Munich.

The project used a multi-method approach and included a qualitative study of interviews (patients, physicians, shared decision making experts) and video analyses of doctor–patient interactions of treatment-planning consultations within the randomised controlled trial. A grounded theory methodology was used to analyse these qualitative data. Results have been published previously (Caspari, 2007) and, therefore, are not the focus of this paper.

### Procedure

We adapted the decision board introduced by Whelan *et al* (1999) as a decision aid for the surgical treatment of primary breast cancer. In addition, two new decision boards were developed: one for the decision of additional chemotherapy in women with hormone-responsive breast cancer (tumour size T1) and one for patients with tumour size T2 or T3 considering preoperative chemotherapy which was offered within the preoperative chemotherapy clinical trial PREPARE at the time the study was conducted. Additional details on the development of the three decision boards, their contents, and pilot testing are described elsewhere (Vodermaier *et al*, 2004).

Staff doctors informed the two research psychologists (CC, JK) who recruited the patients about new patients who had entered the ward and who were eligible for the trial. Randomisation was conducted after the patient gave written informed consent to participate in the trial through the research staff. Random assignment was performed by means of numbered cards in envelopes for the intervention and the control group, and was stratified by age group (<, ⩾60 years). For participants of the intervention arm, the decision board intervention was provided by research psychologists who had previously recruited the patients. The intervention was conducted on the day the patients arrived in the hospital after completion of the baseline questionnaire and before routine planning with the senior physician who did the subsequent surgery usually on the following day. The decision board intervention took about 20 min and contained four steps. First, patients were explicitly informed about options on which to decide upon and the possibility of participating in decision-making (equipoise statement, Elwyn *et al*, 2000). Next, the two treatment options were explained to the patient. In the third step patients' understanding of the treatment options was discussed. Lastly, options were related to the patient's personal situation. The intervention aimed at motivating patients to ask questions during the physician consultation, to participate in treatment planning, to take time for decision making, and eventually to discuss treatment options with family members.

Patients also received an information brochure with the content of the decision board after the intervention, which remained with the patient and provided them with the opportunity to review information they had received during the intervention. Usually, a few hours after the intervention patients had their appointment with the senior physician with whom they planned surgery and further appointments. In the control arm, as in standard care, the appointment with the senior physician is the time point in hospital procedures during which treatment decision-making usually takes place. Hence, participants of the intervention arm received an informational and decisional intervention and a brochure in addition to standard care.

Patients with a strong suspicion of having early breast cancer (tumour size T1) received decision board I concerning surgical decision making (lumpectomy and radiation *vs* mastectomy). Mastectomy decision making also contained information about the possibility of breast reconstruction.

Within this group, women with hormone-responsive breast cancer also received the information booklet about the decision for or against chemotherapy in addition to hormonal therapy containing the same information as its respective decision board. The results of the hormone receptor did not consistently arrive within a time frame that allowed an additional intervention as patients had been generally discharged within 1 week of admission. Consequently, the originally planned additional decision aid intervention in this patient subgroup could not be performed. Eligible patients, therefore, received the information booklet only during their inpatient stay.

Patients with more advanced breast cancer (tumour size T2 or T3) were presented decision board III comparing preoperative chemotherapy followed by surgery *vs* standard therapy (surgery followed by chemotherapy) in the intervention arm. The decision aid for patients with T2 or T3 breast cancer discussed both the question of whether or not to undergo preoperative chemotherapy as well as the consequences of the respective treatment choice on the cosmetic outcome. It was explained that the possibility of breast-conserving therapy depends on the relation between tumour size and breast size and, therefore, breast-conserving therapy may not be possible for women with larger lumps. The decision aid also informed about the possibility of breast reconstruction for these women. Moreover, the decision aid shortly stated the issue of leaving the lump for a few additional months in the breast and that in consequence for some women immediate surgery might be psychologically more beneficial.

### Measures

Patients were assessed with the following instrumentation:

#### Primary outcome variable

*Decisional conflict*. The Decisional Conflict Scale (DCS; O'Connor, 1995) measures patients' uncertainty about which treatment to choose, factors contributing to uncertainty (believing to be uninformed, unclear values, and unsupported in decision making), and perceived effectiveness of decision making. Questions have to be answered on a 5-point Likert scale. Higher scores on the scale or subscales reflect higher decisional conflict, uncertainty, and a less effective choice. The German version of the scale demonstrated subscale and total score internal consistencies in the present sample between 0.73 and 0.94. The scale discriminates between patients who make and those who delay decisions (O'Connor, 1995; Bunn and O'Connor, 1996) and is sensitive to change (O'Connor *et al*, 1998).

#### Secondary outcome variables

*Uptake rates of treatment options.* For participants with supposed early-stage breast cancer (tumour size T1), we assessed the type of surgery (breast-conserving therapy and radiation *vs* mastectomy) and whether additional chemotherapy was chosen in patients with hormone-responsive tumours. Patients with tumour size T2 or T3 were monitored as to whether they opted for preoperative chemotherapy or standard treatment. Information on the patients' treatment was taken from the patients' charts.

*Length of consultation*. Patients were asked about the amount of time the physician spent with them for treatment planning, with the following categories: 5–10, 10–15, 15–25, 25–35, more than 35 min.

*Time point of decision making.* Patients were asked to indicate at what time the treatment decision making took place. The potential options provided were: during treatment-planning session, the same day, the day after, or several days later.

*Patient perception of treatment decision making*. Patients were asked on a five-point scale to what degree the doctor or the patient decided about the treatment with the following categories: physician decided alone, physician decided predominantly, treatment decision making was equally shared between doctor and patient, patient decided predominantly, patient decided alone.

*Perceived involvement in care*. To examine whether the decision aid intervention influenced physician–patient interaction, we applied the German version of the Perceived Involvement in Care Scale (PICS; Lerman *et al*, 1990; Scheibler *et al*, 2004). The PICS consists of three subscales: doctor facilitation of patient involvement, level of information exchange, and patient participation in decision making. At the time of patient recruitment only two subscales of the German version (doctor facilitation, patient information) had adequate psychometric properties (Scheibler *et al*, 2004) and were included in the study. The answering format is a 4-point Likert scale with higher scores reflecting more involvement. The split half reliability coefficients (Spearman–Brown) of the two subscales were 0.84 and 0.79, respectively (Scheibler *et al*, 2004).

*Satisfaction.* To measure satisfaction related to various aspects of the decision making process we used a scale developed by Man-Son-Hing *et al* (1999). The scale consists of six items (Table 1), which were analysed individually in the original report. Questions have to be answered on a 5-point scale.

To assess general patient satisfaction, the German Version of the Client Satisfaction Scale, the ZUF8 (Attkisson and Zwick, 1982; Schmidt *et al*, 1989) was included. The scale consists of eight items with being answered on a 4-point Likert scale. The scale's reliabilities in different samples were between 0.87 and 0.93 and correlated with treatment outcomes (Schmidt *et al*, 1989).

#### Demographic and clinical variables

*Demographic variables*. Data were collected on the women's age, marital status, education level, and employment status.

*Clinical variables*. We assessed tumour size, nodal status, grading, and hormone receptor status.

### Statistical considerations

To detect a medium effect size (*r*=0.3) with a power of 0.80 and a type I error of 0.05 at least 85 patients had to be enrolled in the study (Cohen, 1977). Group comparisons were calculated with independent sample *t*-tests for continuous variables and *χ*^2^-test and Fisher's exact test for categorical variables. The outcome categories of the treatment-specific satisfaction scale (Man-Son-Hing *et al*, 1999) were reduced to two, based on clinical importance (satisfied *vs* undecided or unsatisfied) and also to make sure that there were enough patients in each response category for the analyses. Likewise, the patient rating on the degree of participation in treatment decision making was reduced to three categories: primarily the physician decided, shared decision, and primarily the patient decided. All *P*-values reflect two-tailed tests.

## Results

### Trial retention

**Figure 1:**
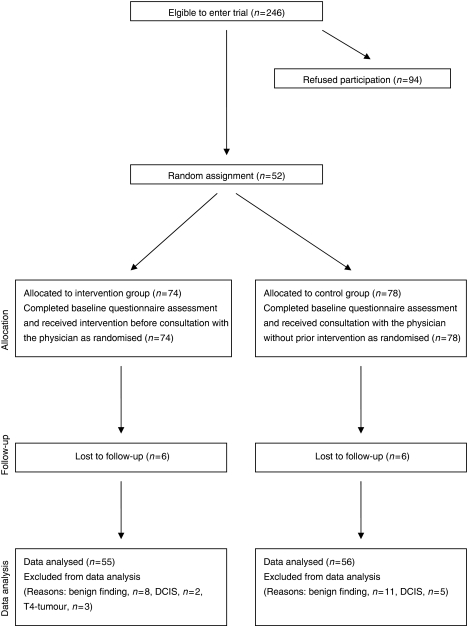
Trial accrual and retention.

Between May 2003 and October 2004 we invited 246 patients to participate, of whom 94 declined (uptake rate of 62.6%). Patient accrual according to the Consort guidelines is presented in Figure 1. The major reasons for declining study participation were preoperative distress associated with filling out a questionnaire shortly after admission (70%), general refusal to fill out questionnaires (10%), involvement in another study therefore not wanting to participate in an additional study (2%), first agreed to participate, but refused after reviewing the questionnaire (4%) or were ineligible because of missing diagnostic findings, outstanding examinations, or preoperative chemotherapy before having filled out the baseline questionnaire (4%). 152 participants were randomised to either the intervention or the control condition. The final sample was 111 patients, of whom 55 were randomly assigned to the intervention group and 56 to the control group. Here, 13 and 16 patients respectively were excluded from the analyses because of *post hoc* ascertainment of not having met the inclusion criteria (benign diagnosis, carcinoma *in situ*, Stage IV-tumour).

### Sample characteristics and success of randomisation

No group differences in terms of demographic and tumour-related variables were found (Table 2). Participants' mean age was 53.5 and 56.9 years, respectively. Seventy-three percent of the sample was married or common law. One third of participants had at least a high school degree and 20% reported graduating from university. About half of the sample was in part or fulltime employment at the time of their diagnosis.

Fifty-nine percent of participants had T1 breast cancer. The remaining had T2 and T3 breast tumours. Eighty-one percent had node-negative breast cancer. As no routine preoperative breast biopsy was offered for early-stage breast cancer patients during the conduct of the study, results from tumour biologic analyses differed somewhat from the pre-randomisation assessment (Table 2).

#### Primary outcome variable

*Decisional conflict*. Overall, participants experienced a modest amount of decisional conflict. No intervention effect emerged on the decisional conflict total scale. Among subscales, a significant group effect, effect size *η*_p_^2^=0.06, on the ‘uninformed’ subscale revealed that patients of the intervention group felt more informed as compared to the control group (Table 3).

#### Secondary outcome variables

*Perceived involvement in care.* No group differences were found in patients' perception of the extent of involvement in the treatment choice (Table 3). Here, 67% of participants reported that they shared the decision-making with their physician, 28% that the physician was more influential on the decision, and 6% found that they themselves were more influential.

Patients reported a moderate to high amount of patient activation by the doctor and their information seeking behaviour, which did not vary as a function of the treatment arm (Table 3).

*Length of consultation.* No time differences emerged in the length of the treatment decision consultation with the physician on patient self-reports. The mean time for the treatment decision making appointment was about 15 min. (Table 4)

*Time point of decision making.* In all 75% of participants reported that the decision was made during the treatment-planning session. Only 12% of patients reported having spent a day or more to decide on a specific option. Groups did not differ in the time frame used for treatment decision making.

*Satisfaction with decision and treatment*. Overall, participants reported high satisfaction with the decision making process and general satisfaction with treatment, which did not vary as a function of the treatment arm.

#### Subgroup analyses

*Uptake rates of treatment options.* In subgroup analyses we tested whether treatment decisions varied as a function of study arm. The decision aid intervention did not influence uptake rates of any of the three treatment options (Table 4). Most patients with T1 breast cancer (91%) opted for breast-conserving therapy. Also, no group differences emerged in uptake rates of chemotherapy in the subgroup of patients with hormone-responsive early breast cancer and decision making towards preoperative chemotherapy in women with more advanced breast cancer. Thirty percent of participants with T1 breast cancer disease decided for chemotherapy in addition to hormone therapy whereas 55% (IG: 62.3% *vs* CG: 46.7%) of patients with more advanced disease decided for preoperative chemotherapy.

## Discussion

Overall, the decision aid intervention in patients with newly diagnosed breast cancer had little impact on the patients' experience of the treatment decision making procedure. The main hypothesis, that participants of the intervention arm were less likely to experience decisional conflict cannot be confirmed. However, on the ‘information’ subscale of the decisional conflict scale which is considered as one of two indicators of decision quality (O'Connor *et al*, 2003), patients who underwent the decision aid intervention reported feeling better informed than participants in the control arm. Effects on informational levels have been also shown in other studies in women with newly diagnosed breast cancer (Sawka *et al*, 1998; Whelan *et al*, 2003, 2004).

No other differences emerged between groups. In line with other studies (Sepucha *et al*, 2000; Whelan *et al*, 2003), no effect was found on the length of the consultation with the senior physician being the main doctor with whom to decide upon the following treatment. Thus, existing data do not support the notion that decision support interventions affect physicians' timing but the doctors' consultation may vary by themes from basic knowledge translation to more individual patient counselling. In this study, however, unblinding of conditions may have resulted in some reactivity of physicians with taking more emphasis on counselling patients in terms of shared decision making in the control group. Nonetheless, a minority of physicians may have been aware of the patients' group status of the trial.

No group differences emerged in patients' self-report of doctors' behaviour. Patients reported the same amount of involvement in care and the same proportion of decision autonomy independent of group membership. Two explanations may be responsible for these results. Independent of the intervention arm, most participants experienced a high degree of participation in treatment decision making. Consequently, treatment decision making counselling may have had, *a-priori*, met shared decision making criteria. On the other hand, the procedures of inpatient counselling, for the majority of patients one day prior to surgery, seem to be inadequate to implement a shared decision making approach. Because shared decision making may have implicitly, become a standard in patient counselling, possibly also activated through the conduct of the trial, an additional effect of the intervention may have been prevented by the inpatient treatment setting. In line with these results, the time point of decision making did not vary as a function of treatment arm as well.

A different interpretation of the data can be derived from the qualitative analysis of video-taped treatment consultation planning sessions with the senior physician during the conduct of the trial (Caspari, 2007). These findings suggest that most patients desire a quick treatment, trust their physicians in the uncertain situation, and do not discriminate between shared decision making and the relationship to their physician. A high level of trust in the physician is experienced as equivalent to participating in treatment decision making. Hence, derived from qualitative data of the trial, patient involvement in treatment decision making does not contradict decision delegation to the physician. It could have been possible that the doctor–patient interaction coloured the evaluation of the treatment decision and may explain lacking group differences of the randomised controlled trial.

Furthermore, no group differences in treatment choice emerged. However, uptake rates of breast-conserving therapy significantly exceeded rates reported in other studies, especially in the control condition (Street *et al*, 1995; Molenaar *et al*, 2004; Whelan *et al*, 2004). Therefore, a ceiling effect may have emerged on this variable. Other evidence demonstrated that the endorsement of breast loss and fear of recurrence predicted either uptake of lumpectomy or mastectomy and not the introduction of decision aids (Molenaar *et al*, 2004). With regards to chemotherapy, about 30% of patients with early breast cancer decided for adjuvant treatment. Although one study reported no effect on adjuvant treatment decision making either (Whelan *et al*, 2003), a decrease in adjuvant chemotherapy uptake in the group of patients who received a decision aid has been reported (Peele *et al*, 2005; Siminoff *et al*, 2006). Owing to the size of the subsample results on uptake rates of preoperative chemotherapy decision making have to be considered as preliminary. It would be interesting if this trend found in subgroup analyses could be replicated in a larger study with more power to detect a significant effect. As outlined earlier, however, differences in treatment choice should not be considered as an indicator of informed decision making or decision aid efficacy.

Satisfaction with the decision making process and general patient satisfaction with care was high and did not differ between groups. Though physicians involved in the patients' treatment did not see patients' assessment of treatment satisfaction, ratings were not blind to the research psychologists who performed the intervention. This may have lead to social desirability with regard to the evaluation of the decision aid intervention but may have had little impact on ratings of satisfaction with treatment. Variance constriction because of a ceiling effect may have contributed to the lack of a group difference. Results are in line with other studies, which reported null effects of decision aids on satisfaction judgements (Sawka *et al*, 1998; Whelan *et al*, 1999), whereas some studies demonstrated short (Whelan *et al*, 2004) or long-term effects (Molenaar *et al*, 2001). Discrepant findings may be related to individual differences in cognitive processing of the decision making phase on which the decision aids also have an impact. For most women with breast cancer the treatment-planning phase is a highly remembered time point in the disease trajectory. Many women feel relief post-treatment, experience few decisional conflict and satisfaction with treatment. On the other hand, some patients experience ongoing doubts and uncertainty whether they have undergone the ‘right’ treatment or even experience feelings of regret for not having taken some aspects into consideration they value important in retrospect (Lantz *et al*, 2005; Sheehan *et al*, 2007). The degree to which patients experience high uncertainty or regret could both depend on situation-specific experiences how their treatment decision making occurred as well as on a rather stable personality trait of frequent rumination. These patient characteristics may interact with decision aid interventions on outcome measures.

The study's findings must be considered in relation to some limitations. A notable proportion of patients invited to participate in the study declined and a considerable number of post-randomisation exclusions occurred due to the fact that an invasive breast cancer diagnosis was not confirmed prior to randomisation by means of a lack of preoperative breast biopsy. As we wanted to assess the effect of the intervention in breast cancer patients, we decided to only include participants with invasive breast cancer and excluded patients with benign disease, ductal carcinoma *in situ*, and metastatic diseases from the analyses. Patients with benign findings experience a huge relief, which may colour the evaluation of the decision aid intervention whereas patients with DCIS have less treatment options and patients with metastatic disease are facing completely different decisions and challenges.

Furthermore, the results of the study are limited to one centre and the study was not powered to detect effects on treatment decision making in subgroup analyses, especially with regards to detecting intervention effects on the decision towards preoperative chemotherapy. Also, randomisation performed by a computer is a more robust method than the random assignment by research staff as conducted in this study.

Except for treatment decision making, all outcome variables were based on self-report measures. Specifically, patients' reports on the length of the consultation with the senior physician in which the decision was discussed or even finalised may have involved some inaccuracy of measurement. However, it was not possible within the study to obtain independent time assessments.

A strength of the current study is its representativeness. The sample did not differ in terms of demographic and tumour-related characteristics from the regional epidemiological tumour registry database of breast cancer patients for southern Bavaria (Engel *et al*, 2005), suggesting the study's generalisability with regards to the study sample. In terms of the treatment setting the findings are generalisable to university hospital treatment for breast cancer. The introduction of decision aids in general hospitals may likely differ with regards to lower standards of patient counselling and consequently may heighten the importance of decision aids for information provision.

In conclusion, this study showed a small effect of a decision aid intervention in newly diagnosed breast cancer patients in the hospital inpatient setting on their subjective level of information. These almost null findings may be related to a pre-existing high standard of shared decision making realisation in the physicians' guidance of patients or circumstances in the inpatient treatment setting that hinder achieving further improvements through the introduction of decision aids. Hence, the study suggests that contextual factors (i.e., inpatient treatment, short time frame for decision making) may explain low decision aid effectiveness.

As decision aid interventions in the treatment of cancer patients have demonstrated rather small effects across diseases, future research may address the role of contextual factors that promote the implementation and effectiveness of shared treatment decision making. Moreover, the shared decision making paradigm suggests that shared medical decision making involves favourable effects for all patients. No randomised controlled trial to date examined the role of patients' decisional preferences on decision aid effectiveness. Hence, offering decision aid interventions to subgroups of patients who state a strong preference for shared decision making may result in larger effect sizes for these kinds of tools. The examination of individual differences, therefore, may identify subgroups of patients who may derive more benefits from the introduction of decision aids than their application across all patients.

## Figures and Tables

**Table 1:** Impact on satisfaction with decision and treatment

**Variable (range)**	**Intervention group**	**Control group**	***χ*^2^/Fisher's test**	***P*-value**
*Physician helped me to understand results*				
Yes	49 (92.5%)	53 (94.6%)	0.22	0.71
No	4 (7.5%)	3 (5.4%)		
				
*Physician understood what is important to me*				
Yes	47 (88.7%)	50 (90.9%)	0.15	0.70
No	6 (11.3%)	5 (9.1%)		
				
*Physician answered all questions*				
Yes	47 (88.7%)	51 (92.7%)	0.53	0.52
No	6 (11.3%)	4 (7.3%)		
				
*Satisfied with involvement in decision making*				
Yes	44 (83.0%)	45 (83.3%)	<0.01	0.97
No	9 (17.0%)	9 (16.7%)		
				
*Satisfied with physician's involvement*				
Yes	36 (75.0%)	36 (76.6%)	0.03	0.86
No	12 (25.0%)	11 (23.4%)		
				
*Satisfied with process*				
Yes	42 (89.4%)	50 (92.6%)	0.32	0.73
No	5 (10.6%)	4 (7.4%)		
ZUF-8 (4–32)	29.08 (2.99)	28.67 (2.86)	0.71	0.48

**Table 2:** Descriptive statistics [M(s.d.) and *n*(%)] for demographic and tumor-related variables

**Variable**	**Intervention group**	**Control group**	**t/*χ*^2^/Fisher's test**	***P*-value**
Age (n=111)	53.53 (11.67)	56.86 (10.10)	−1.61	0.11
				
*Married/cohabiting (*n=*111)*				
Yes	40 (72.7%)	41 (73.2%)	0.003	0.95
No	15 (27.3%)	15 (26.8%)		
				
*High school (*n=*111)*				
Yes	18 (67.3%)	17(69.6%)	0.07	0.79
No	37 (32.7%)	39 (30.4%)		
				
*University degree (*n=*107)*				
Yes	13 (24.5%)	9 (16.7%)	1.01	0.31
No	40 (75.5%)	45 (83.3%)		
				
*Employed (*n=*110)*				
Yes	27 (50.0%)	25 (44.6%)	0.32	0.57
No	27 (50.0%)	31 (55.4%)		
				
*Tumour size (*n=*104)*^a^				
T1	30 (58.8%)	31 (58.5%)	0.26	1.00
T2	19 (37.3%)	19 (35.8%)		
T3	2 (3.9%)	3 (5.7%)		
				
*N0 (*n=*104)*				
Yes	45 (88.2%)	40 (75.5%)	2.84	0.09
No	6 (11.8%)	13 (24.5%)		
				
*Hormone receptor (*n=*110)*^b^				
Positive	48 (88.7%)	49 (90.4%)	0.09	0.78
Negative	7 (11.3%)	6 (9.6%)		
				
*Grading 3 (*n=*108)*				
Yes	13 (24.1%)	14 (25.9%)	0.05	0.82
No	41 (75.9%)	40 (74.1%)		

a Actual tumour size was different from pre-randomisation assessment which resulted in a higher proportion of patients clinically classified with T1 breast cancer than after tumour biologic analyses.

b Numbers are different from Table 4 (limitation to T1 breast cancer) whereas here information on the hormone receptor status is given for the whole sample.

**Table 3:** Impact on decisional conflict

**Variable (range)**	**Intervention group**	**Control group**	***t*-test**	***P*-value**
DCS uncertainty (1–5)	2.08 (0.97)	2.20 (0.90)	−0.65	0.52
DCS uninformed	1.88 (0.63)	2.17 (0.79)	−2.01	0.048
DCS unclear values	1.83 (0.62)	1.99 (0.79)	−1.16	0.25
DCS unsupported	1.65 (0.65)	1.84 (0.63)	−1.49	0.14
DCS ineffective choice	2.13 (0.83)	2.40 (0.80)	−1.68	0.10
DCS total	1.82 (0.59)	1.99 (0.62)	−1.36	0.18
PICS doctor facilitation (1–4)	2.65 (0.66)	2.72 (0.67)	−0.56	0.58
PICS patient information	3.04 (0.74)	3.09 (0.73)	−0.35	0.73

**Table 4:** Impact on treatment decision making

**Variable**	**Intervention group**	**Control group**	**t/*χ*^2^/Fisher's test**	***P*-value**
*Breast-conserving therapy (*n=*80)*^a^				
Yes	37 (94.9%)	36 (87.8%)	2.01	0.27
No	2 (5.1%)	5 (12.2%)		
				
*Chemotherapy (*n=*74)*^a^				
Yes	11 (31.4%)	11 (28.2%)	0.09	0.80
No	24 (68.6%)	28 (71.8%)		
				
*Pre-operative chemotherapy (*n=*31)*^a^				
Yes	10 (62.5%)	7 (46.7%)	0.78	0.48
No	6 (37.5%)	8 (53.3%)		
				
*Length of consultation (*n=*107)*				
5–10 min	6 (11.3%)	5 (9.3%)	1.03	0.91
10–15 min	17 (32.1%)	19 (35.2%)		
15–25 min	15 (28.3%)	14 (25.9%)		
25–35 min	7 (13.2%)	5 (9.3%)		
Above 35 min	8 (15.1%)	11 (20.4%)		
				
*Decision making (*n=*97)*				
During treatment planning session	36 (75.0%)	38 (77.6%)	0.64	0.93
The same day	6 (12.5%)	5 (10.2%)		
The day after	2 (4.2%)	3 (6.1%)		
Several days later	4 (8.3%)	3 (6.1%)		
				
*Patients' perception of decision making (*n=*107)*				
Primarily the physician	14 (26.4%)	16 (29.6%)	0.81	0.72
Shared	35 (66.0%)	36 (66.7%)		
Primarily the patient	4 (7.5%)	2 (3.7%)		

a Different subsamples were tested. Percentages reflect decisions within decisional options patients' were provided and are somewhat different from tumour biological results, which were provided later.
